# The Role of Cardiovascular Imaging in the Evaluation of Rheumatic and Neuromuscular Disorders

**DOI:** 10.3390/jcm9113614

**Published:** 2020-11-10

**Authors:** Sophie I. Mavrogeni

**Affiliations:** 1 Onassis Cardiac Surgery Center, Department of Cardiology, 17674 Athens, Greece; sophie.mavrogeni@gmail.com; 2 Department of Pediatrics, National and Kapodistrian University of Athens, 17674 Athens, Greece

Autoimmune rheumatic diseases (ARD) and neuromuscular disorders can affect a number of organs. ARDs are characterized by the impairment of adaptive immunity, such as the production of autoantibodies and autoreactive T cells [1]. Cardiovascular abnormalities in ARDs are due to various pathophysiologic processes, such as myocardial/vascular inflammation, accelerated atherosclerosis, myocardial ischemia due to micro- of macro-vascular disease, abnormal coronary vasoreactivity, and/or myocardial fibrosis [2,3]. ARD patients may present with valvular, myocardial, and pericardial inflammation, coronary artery disease, vasculitis, myocardial fibrosis, heart failure, as well as pulmonary arterial hypertension. Unfortunately, CVD in ARD may be asymptomatic or characterized by subtle, usually underestimated symptoms. When cardiac disease becomes clinically overt, it is indicative of advanced myocardial impairment and carries a poor prognosis [4]. However, the early, aggressive application of targeted anti-rheumatic treatments may aid in the significant reduction of disease-associated CVD mortality. The life expectancy in ARD still remains lower, compared with the general population [5], mainly due to CVD [6,7,8,9,10]. ARDs associated with cardiovascular involvement include: (1) rheumatoid arthritis and the spondyloarthropathies; (2) systemic lupus erythematosus; (3) systemic vasculitides; (4) inflammatory myopathies; (5) systemic sclerosis; (6) mixed connective tissue disease (MCTD); and (7) sarcoidosis (SRC) [6,7,8,9,10].

The most common reasons for “missing” early CVD in ARD include: (a) atypical clinical presentation; (b) ignorance of CVD which is attributed to patient’s general symptoms; and (c) lack of sensitivity of the currently used noninvasive, diagnostic techniques in cardiology to detect early cardiovascular lesions occurring in ARD. 

Neuromuscular disorders include a spectrum of various diseases that may affect skeletal muscles and myocardium in both patients and asymptomatic carriers [11]. The most common are Duchenne (DMD) and Becker muscular dystrophy (BMD). DMD is a X-linked recessive disorder that affects 1 in 3500 males [12,13], caused by mutations in the dystrophin gene that leads to the reduced or deficient synthesis of dystrophin, a part of the dystrophin-glycoprotein complex (DGC) [14]. Dystrophin is one of the largest proteins in the body and it is expressed not only in skeletal but also in cardiac muscle and in the brain. Although DMD is described as a primary degenerative condition of the skeletal muscles, it has also detrimental effects on the heart [11]. Female carriers of DMD are usually free of skeletal muscle symptoms, although 30% develop some kind of skeletal muscle problems and about 10% of these will also develop cardiac involvement [15]. BMD is a milder form, also caused by mutations in dystrophin gene. BMD patients can usually walk and have a nearly normal life expectancy. However, they have a 50% chance of developing cardiac disease [16].

Our aims in this special issue of JCM are: (a) to present the role of noninvasive imaging modalities in the early detection of cardiovascular involvement in this diseases; (b) to increase awareness of internists, cardiologists, rheumatologists, and neurologists regarding cardiac disease in these patients; and (c) to discuss potential risk stratification and treatment changes according to the results of various imaging modalities.

We hope that this issue of our journal will enrich your diagnostic ability, will bring you closer to the pathophysiology of these diseases and facilitate your diagnostic and therapeutic approach.
